# The diversity, dynamics, and culturability of bacterial and fungal communities present in warm-season pasture grass seeds

**DOI:** 10.3389/fmicb.2025.1621463

**Published:** 2025-06-25

**Authors:** Rens R. T. van Essen, Jatinder Kaur, Tongda Li, Ross C. Mann, Tim I. Sawbridge

**Affiliations:** ^1^Department of Energy, Environment and Climate Action, Agriculture Victoria, AgriBio, Centre for AgriBioscience, Melbourne, VIC, Australia; ^2^School of Applied Systems Biology, La Trobe University, Melbourne, VIC, Australia

**Keywords:** warm-season pasture grasses, bacterial profile, fungal profile, culturability, metagenomics, seed microbiome

## Abstract

A rapidly changing climate has resulted in increasing challenges for farmers. This has led to an increase in demand for beneficial microbes to help fight these challenges faced by farmers, improving crop production under harsh conditions. Increasing temperatures caused by the changing climate will also affect the dairy industry in temperate climates around the world. This has resulted in an increasing importance of warm-season pasture grasses to fill the feed gaps left by the affected temperate grasses. In this study, we assessed the microbial communities present in commercially available warm-season pasture grass seeds. We utilised amplicon metagenomics to profile and compare the bacterial and fungal communities of seeds from three different genera of warm-season pasture grasses. Microbial isolations have also been performed to assess the culturability of the seed microbiome. Significant differences in drivers of bacterial and fungal communities within warm-season pasture grass seeds were observed. In addition, most of the bacteria present in high abundance were found to be culturable, while a relatively lower percentage of abundant fungi were culturable. Analysis of the bacterial communities showed considerable variation between different distributors, possibly driven by differing seed processing methods. This variation indicates that the bacterial communities could be manipulated by providing different bacteria to the seed to promote plant growth under different conditions. In contrast, the fungal communities were more strongly driven by the genetics of the respective host genera. This suggests that differences in fungal strain levels could be exploited for modification of fungal microbiome effects.

## Introduction

The dairy industry, worth 860 billion US$ in 2024, is one of the largest global agricultural industries (Hill, 2024). In many parts of the world, the dairy industry is undergoing rapid intensification and growth to meet the growing demand for dairy products (OECD/FAO, 2024). However, the changing environment, with increasing temperatures and changes in rainfall patterns, has a growing impact on the agricultural industry (Alhassan and Hadwen, 2017; Matzrafi, 2019). These changes result in a reduction in yield for crops while encouraging weed and pest proliferation (Li et al., 2020). In addition, changing weather events caused by global warming create new and major challenges for farmers (Sharma et al., 2022). To increase crop yield, farmers have been relying on agricultural chemicals, which have a high impact on the environment in turn (Zhang et al., 2018). This has led to legislation being put in place to restrict farmers from using these chemicals (Nicolopoulou-Stamati et al., 2016). To fight these challenges in an environmentally friendly way, the use of biological fertilisers and bioprotectants has increased in popularity (Nosheen et al., 2021).

In Australia, the largest proportion of the dairy industry is centred around Victoria, where the cooler climate-adapted temperate grasses are used in the dairy industry (Godfrey et al., 2022). While, in south-eastern Queensland, eastern New South Wales, and south-western Australia, warm-season grasses are very successful as the temperatures in these areas are significantly higher than Victoria (O’Sullivan, 2013; Lattimore and McCormick, 2012; Moore et al., 2006). With the climate changing, these higher temperatures could soon reach Victoria, and the success of these grasses in warmer regions is a great prospect for Victoria and other cooler areas around the world (Moser et al., 2004). Commercially sold warm-season pasture grasses available in Australia include *Cenchrus clandestinus* (Kikuyu grass), *Chloris gayana* (Rhodes grass), and *Paspalum* sp. (Bahia and Dallis grass). All three genera flourish in subtropical and dry climates contain a high nutritional value, do not have many pest or pathogen issues, and they spread very quickly to cover large areas of land (Clark, 2007; Fulkerson et al., 2021; Loch et al., 2004; Jiang et al., 2022; Da Motta et al., 2016). To aid the dairy industry in Australia under changing conditions, understanding the microbiome of warm-season grass seeds may provide access to beneficial seed-born microbes, as has been shown in temperate pasture grasses (Li et al., 2020). Isolating potential beneficial microbes from seeds may allow the application of these microbes to crops as seed treatments.

The aim of this study was to examine microbial communities associated with seeds of commercially available warm-season pasture grasses and possible factors influencing these communities and to assess the culturability of these microbes. This was done through metagenomic sequencing of the 16S and Internal Transcribed Spacer (ITS) regions of the bacterial and fungal ribosomal RNA (rRNA), respectively. This will give an insight into dominant bacterial and fungal taxa within the communities of warm-season grass seeds. Comparisons of these profiles between host genera and suppliers for both bacteria and fungi will allow the identification of possible drivers of the community structure within these seeds. To assess the culturability of the microbes present in warm-season pasture grass seeds, bacterial and fungal isolations were performed using two media, and the identity of these isolates was compared to the microbial profiles. This will also provide a resource of microbes to test for beneficial functions in warm-season pasture grasses.

## Materials and methods

### Warm-season grass seed germination and DNA extraction

Seeds from three different genera of warm-season grasses were sourced from four different distributors (Table 1). Two cultivars (cv. Whittet and Acacia plateau) of *Cenchrus clandestinus* were obtained from McKays seeds, Anco seeds, and Williams Group Australia, with all seeds being produced in north-eastern New South Wales (NSW). Six cultivars of *Chloris gayana* (Katambora, Mariner, Tolgar, Endura, Callide, and Excel) were sourced from McKays seeds (produced in southeast Queensland), Barenbrug (produced in northern Queensland), and Williams Group Australia (produced in southeast Queensland and north-eastern NSW). *Paspalum dilatatum* seeds were acquired from Williams Group Australia (produced in the coastal area of NSW), while *P. notatum* seeds were obtained from McKays seeds, being the only seed batch in the collection produced outside Australia, specifically in Arizona, USA.

**Table 1:** List of warm-season grass seed batches used in this study, including species name, common name, distributors, cultivars, assigned seed batch names, and geographical location of seed production.

Species	Common name	Distributor	Cultivar	Seed batch name	Geographical location
*Cenchrus clandestinus*	Kikuyu grass	McKays seeds	Whittet	CcWM	North-eastern New South Wales
		Anco seeds	Whittet	CcWA	North-eastern New South Wales
		Williams group	Whittet	CcWW	North-eastern New South Wales
			Acacia Plateau	CcAW	North-eastern New South Wales
*Chloris gayana*	Rhodes grass	McKays seeds	Katambora	CgKM	South-eastern Queensland
		Barenbrug	Katambora	CgKB	Northern Queensland
			Mariner	CgMB	Northern Queensland
			Tolgar	CgTB	Northern Queensland
			Endura	CgEB	Northern Queensland
		Williams group	Katambora	CgKW	South-eastern Queensland
			Callide	CgCW	North-eastern New South Wales
			Excel	CgEW	North-eastern New South Wales
*Paspalum dilatatum*	Dallis grass	Williams group		PdW	Arizona, USA
*Paspalum notatum*	Bahia grass	McKays seeds	Pensacola	PnM	Eastern New South Wales

Seeds from different batches were washed for 30 s in sterile distilled water (SDW), and a total of 30 washed seeds were placed in three petri dishes (10 seeds per plate) containing three sterile filter papers moistened with 3 mL of SDW. As seed germination and growth rates are different across species, they were harvested at different time points to ensure similar-sized seedlings were used. *C. clandestinus* seeds were harvested 10 days after planting (DAP), *C. gayana* seeds 14 DAP, and *Paspalum* sp. seeds 21 DAP. For each seed batch, 16 seedlings of 4–6 cm in length, which were not visually contaminated with overgrowing microbes and were not close to any contaminated seedlings, were harvested and added to 1.2 mL collection tubes (Qiagen cat# 9560) containing a single sterile stainless-steel bead (3 mm). The collection microtubes were stored at −80°C and subsequently used for DNA extraction. DNA was extracted using the QIAGEN MagAttract 96 DNA Plant Core kit (Cat# 67163) as per the manufacturer’s instructions.

### Amplicon sequencing of bacteria and fungi in seeds

Amplicon libraries were prepared following the 16S Metagenomic Sequencing Library Preparation guide from Illumina, with minor modifications. The amplicon PCRs were conducted using primers 515F (Forward, 5′-GTGCCAGCMGCCGCGGTAA-3′) and 806R (Reverse, 5′-GGACTACNVGGGTWTCTAAT-3′), targeting the V4 region of the 16S rRNA gene (Caporase et al., 2011), and 58A2 (Forward, 5′-ATCGATGAAGAACGCAG-3′) and ITS4-KYO1 (Reverse, 5′-TCCTCCGCTTWTTGWTWTGC-3′), targeting the ITS-2 rDNA gene region (Bokulich and Milles, 2013). Peptide nucleic acid (PNA) PCR blockers (50 μM) from PNA BIO Inc. were added to reduce the amplification of 16S rRNA gene sequences derived from the plant organelle genomes (Lundberg et al., 2013; Wagner et al., 2016). The PCR was performed following the Illumina protocol, except for the 16S amplicon PCR, where an extra step of PNA clamping was included at 75°C for 10 s. The index PCR was conducted using the Illumina Nextera XT indices to apply combinatorial dual index pairs for sample identification, as per the instructions in the 16S Metagenomic Sequencing Library Preparation guide. Libraries were quantified using a High Sensitivity DNA (HSD) 1000 ScreenTape on TapeStation (Agilent technology cat# 5067–5584) to ensure that amplification of the target had occurred. After each PCR, a cleanup was performed using Promega ProNex Size-Selective Purification system beads (Cat# NG2001). The amplified samples were subsequently normalised using a SequalPrep Normalization Plate (96) kit (Applied Biosystems, cat# A10510-01). Samples were pooled and sequenced following the Illumina MiSeq System Denature and Dilute Libraries Guide.

### Amplicon analysis

Sequencing data of the 16S amplicon was analysed through a QIIME 2 pipeline (version 2022.2). Raw reads were trimmed and merged using the plugin “PANDAseq” (Masella et al., 2012). The merged reads were imported by creating a manifest as single-read FASTQ sequences. The remaining mitochondrial and chlorophyll reads were removed from the data (Bolyen et al., 2019), and the QIIME 2 plugin “DADA2” was used for denoising, assembly of a feature table, and filtering of reads. The QIIME 2 “feature classifier” plugin (Bokulich et al., 2018) was used for the taxonomic assignment of amplicon sequence variants (ASVs) from the SILVA SSU database 138 (released 16 December 2019). The ITS amplicon data were analysed through a modified QIIME 2 (version 2022.2) pipeline. Raw reads were trimmed and truncated using the QIIME 2 “cutadapt trim-paired” plugin (Martin, 2011), and the “DADA2” plugin was used to join and denoise the reads (Callahan et al., 2016). The QIIME 2 “feature classifier” plugin (Bokulich et al., 2018) was used for the taxonomic assignment of ASVs from the UNITE database (version 8, 2020). Both the 16S and ITS data were further analysed though the QIIME 2 plugin “phylogeny,” which was used to generate rooted and unrooted phylogenetic trees through MAFFT and FastTree 2 (Katoh and Standley, 2013; Price et al., 2010). An ASV table was created through Biological Observation Matrix (BIOM) for further analysis. The analysis included preparing a 100% bar chart and a heatmap with a dendrogram (Pearson correlation) showing the most abundant ASVs within the profiles of 14 seed batches. For these figures, the ASVs within each seed batch, after combining the 16 replicates with an abundance of at least 10%, were selected as abundant ASVs (aASVs) and visualised using OriginPro 2020 (64-bit, version 9.7.0.188). In addition, the ASV table was used to prepare principal coordinate analysis (PCoA) plots, which were generated using RStudio (version 2024.04.2) using the R package vegan (Oksanen et al., 2017). These plots were prepared based on the Bray–Curtis distance matrix, utilising the package DESeq2 using the normalised reads, and the distance was statistically quantified through PERMANOVA tests. The DADA2 feature table and filtered reads were also used for Shannon’s alpha diversity analysis in QIIME 2 based on the Kruskal–Wallis test, which includes the H-value (Kruskal–Wallis test statistic), *p*-value, and Q-value (false discovery rate-corrected p-value, through Benjamini–Hochberg correction). From this analysis, the Shannon entropy values were extracted and visualised in overlapping boxplots within OriginPro 2020 (64-bit). Venn diagrams showing the 10% most abundant microbes after combining the 16 replicates were created for each seed batch in Rstudio using the R package VennDiagram.

### Isolation and storage of bacterial and fungal microbes

Five seedlings of approximately 4 to 6 cm in size were pooled into a sterile 1.7 mL Eppendorf tube with two sterile stainless-steel beads (3 mm). To each tube containing five seedlings, 300 μL of 1× phosphate-buffered saline (PBS) was added and subsequently ground twice using QIAGEN TissueLyser II at 25 Hz for 30 s following the manufacturer’s protocol. After grinding, the tubes were spun at 6000 revolutions per minute (RPM) for 5 min and the supernatant was transferred to a clean, sterile 1.7 mL Eppendorf tube. From this stock tube, serial dilutions (10^−1^, 10^−2^, 10^−3^, 10^−4^, and 10^−5^) were prepared. The dilutions were plated on solid substrate media, R2A (OXOID, 18.1 g/L) for bacterial growth and ½ PDA (OXOID, 19.5 g/L) with 100 mg/L tetracycline for fungal growth. To isolate bacteria, 80 μL of the stock and 40 μL of the dilutions 10^−1^, 10^−2^, 10^−3^, 10^−4^, and 10^−5^ were plated. To isolate fungi, 80 μL of the stock and 40 μL of the dilutions 10^−1^ and 10^−2^ were plated. Depending on the culture development, individual bacteria and fungi were picked after 1 day to 2 weeks of growth and streaked on fresh media to obtain pure isolates. The cultures were then allowed to develop for a further 4 days (bacteria) and 7 days (fungi), respectively, to ensure that pure bacterial colonies and fungi could be picked for their long-term storage at −80°C. For each bacterial isolate, an overnight liquid culture of 20 mL of nutrient broth (NB) was prepared with a single colony and grown at 28°C while shaking at 150 RPM. From this overnight liquid culture, 750 μL was added to 750 μL of 40% glycerol in a screwcap 1.5 mL cryotube, which was flash-frozen with liquid nitrogen and stored at −80°C. For fungal isolates, stock cultures were prepared by cutting agar into 5 mm^2^ samples from cultures that had sporulated. Stock cultures were put into a 1.5-mL screwcap Eppendorf tube with 750 μL of 20% glycerol and also into another screwcap Eppendorf tube containing 750 μL of SDW. These tubes were put into tube holders containing isopropyl alcohol to slowly freeze the fungal isolates at −80°C. After 24 h, the tubes were moved into a storage box and kept at −80°C.

### Sequence-based identification of bacterial and fungal isolates

To identify bacteria, a colony PCR was performed directly on single bacterial isolates that were picked from media and suspended into 50 μL SDW. The bacterial samples were mixed by stirring and subsequently incubated in a thermocycler for 10 min at 99°C. The samples were centrifuged at 4000 RPM for 5 min, and 2 μL of the supernatant was used as a template for the PCR. The PCR was performed according to the protocol described for the OneTaq 2X Master Mix with Standard Buffer (NEBiolabs, Cat# M0482S) with 2 μL of template. The PCR contained OneTaq 2X Master Mix, 10 μM 27F forward primer (5′-AGAGTTTGATCMTGGCTCAG-3′), and 10 μM 1492R reverse primer (5′-TACGGYTACCTTGTTACGACTT-3′) to target the entire 16S rRNA region (Frank et al., 2008). For the fungal isolates, mycelia were collected and added to 100 μL of 50 mg/mL Chelex 100 resin and heated to 95°C for 30 min. This mixture was subsequently centrifuged at 4000 RPM for 5 min, and 5 μL of the supernatant was used as template. The PCR for the ITS amplicon contained OneTaq 2X Master Mix, 10 μM ITS1F forward primer (5′-CTTGGTCATTTAGAGGAAGTAA-3′) and 10 μM ITS4 reverse primer (5′-TCCTCCGCTTATTGATATGC-3′) to target the ITS1 and ITS2 rDNA regions (Manter and Vivanco, 2007). All samples were visualised on a 2% agarose gel, which ran for 30 min at 100 volts, to check the amplification as well as the concentration of the PCR products. Suitable PCR products were sent to Macrogen, South Korea, for sequencing.

### Sequencing analysis of bacterial and fungal isolates

The sequencing results received from Macrogen were initially analysed through the software Geneious Prime (version 2022.2.2). The ends of the raw reads were trimmed, and the forward and reverse reads were aligned through a *de novo* assembly. The sequences were compared to the 16S and ITS sequences from the ASV tables created in the 16S and ITS analysis of the profiling experiment. This was done by using the QIIME 2 plugin “metadata tabulate” to retrieve the sequences of the profiles. The consensus sequences were subsequently used to align to a reference database comprised of all the QIIME 2 ASVs. The assigned consensus reads were compared to the profiling reads, and two box charts were prepared in OriginPro 2020 (64-bit, version 9.7.0.188) to visualise the percentage of profiles coming from culturable ASVs.

## Results

### Microbial comparison of warm-season grass seeds

#### Sequencing data

Sequencing of the bacterial and fungal seed microbiome was done over four different Illumina MiSeq runs. After quality filtering, which included removing low-frequency sequences and chimeric sequences, a total of 5,386,247 reads over the four sequencing runs were left. After similar quality filtering for the fungal communities, there were a total of 18,820,966 sequences left over the four sequencing runs. ASVs were subsequently assigned using the Qiime2 pipeline, resulting in a total of 972 bacterial ASVs and 3,972 fungal ASVs distributed over the 14 different seed batches after combining the results of all 16 replicates. This resulted in an average of three abundant ASVs within the bacterial communities and an average of two abundant ASVs within the fungal communities. An overview of the numbers is shown in Table 2. The full ASV tables for both the 16S amplicon and the ITS amplicon can be found in Supplementary Data.

**Table 2:** Sequencing results from the 16S and ITS amplicon sequencing runs.

Seed batches	Bacterial sequences						Fungal sequences					
	Input	Filtered	Denoised	Non-Chimeric	ASVs	aASVs	Input	Filtered	Denoised	Non-Chimeric	ASVs	aASVs
CcAW	218,135	95,452	94,820	94,612	119	4	1,189,745	843,459	842,123	798,710	114	5
CcWW	207,504	88,711	88,295	87,203	44	3	1,474,100	1,081,794	1,080,227	1,018,051	94	4
CcWA	1,370,021	1,100,443	1,094,859	1,065,125	392	5	3,078,956	2,592,170	2,588,979	2,438,092	332	6
CcWM	398,146	242,521	241,279	238,315	148	5	877,502	610,814	609,821	589,752	93	4
CgCW	353,210	152,562	152,007	151,692	94	2	1,311,130	1,123,505	1,121,578	1,093,044	386	3
CgEW	290,416	136,925	136,091	135,600	99	2	1,204,786	1,031,550	1,030,197	1,000,109	248	4
CgKW	389,205	169,832	169,506	168,985	53	2	1,032,576	872,492	870,938	838,683	264	1
CgEB	504,082	235,203	234,483	233,383	125	3	1,136,457	953,773	952,453	919,388	266	4
CgKB	200,448	94,263	93,997	93,598	55	3	1,227,198	1,044,417	1,042,574	1,016,767	247	1
CgMB	409,737	190,487	189,731	188,199	95	2	1,850,724	1,528,328	1,526,301	1,430,615	347	2
CgTB	1,673,818	1,283,192	1,279,111	1,250,086	291	2	2,891,932	2,550,316	2,547,001	2,430,373	598	3
CgKM	372,620	212,295	211,194	209,989	171	3	1,609,348	1,171,028	1,168,843	1,105,634	422	5
PnM	207,029	111,142	110,533	110,388	67	4	2,146,059	1,617,514	1,614,389	1,534,898	350	3
PdW	1,609,939	1,239,725	1,235,901	1,221,297	265	5	3,377,615	2,794,242	2,790,141	2,606,850	211	7

A total of 5,386,247 bacterial sequences and 18,820,966 fungal sequences resulted in 972 bacterial ASVs and 3,972 fungal ASVs.

#### Bacterial communities

**Figure 1:**
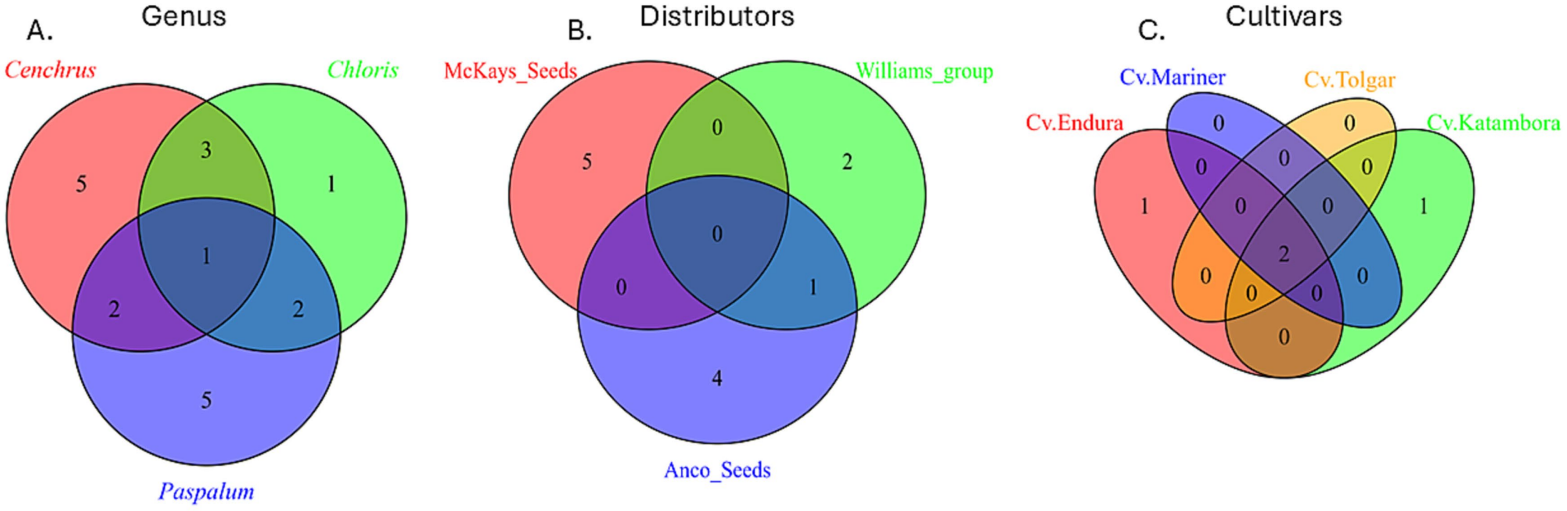
Venn diagram of bacterial communities. **(A)** Comparing the abundant ASVs (aASVs) of three different genera. **(B)** Comparing the aASVs of three different distributors of *Cenchrus clandestinus* from the cultivar Whittet. **(C)** Comparing the aASVs of four different *Chloris gayana* cultivars from the distributor Barenbrug.

**Figure 2:**
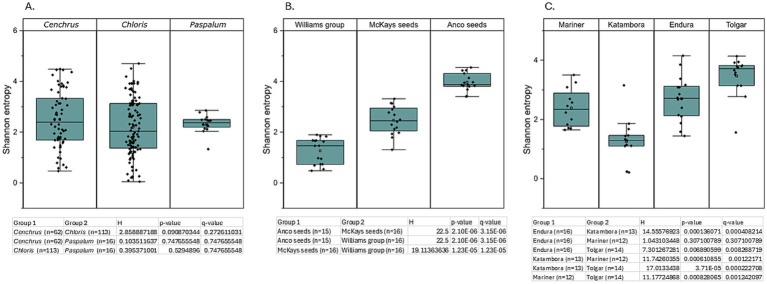
Shannon’s alpha diversity analysis based on pairwise Kruskal–Wallis test between bacterial communities, including the H-value (Kruskal-Wallis test statistic), *p*-value (raw *p*-value from test), and Q-value [false discovery rate-corrected *p*-value (Benjamini–Hochberg correction)] comparing different parameters. **(A)** Shannon’s alpha diversity test comparing three different genera. **(B)** Shannon’s alpha diversity test comparing three different distributors of *Cenchrus clandestinus* from the cultivar Whittet. **(C)** Shannon’s alpha diversity test comparing four different *Chloris gayana* cultivars from the distributor Barenbrug.

The Venn diagram shows the bacterial abundant ASVs (aASVs) on the genus level after combining all 16 replicates used for profiling. These abundant bacteria are present at an abundance of at least 10% within the entire communities of the three genera (Figure 1A). This figure shows a total of 11 different aASVs within four *C. clandestinus* seed batches, seven different aASVs within eight *C. gayana* seed batches, and 10 different aASVs within two *Paspalum* seed batches. Between *C. gayana* and *C. clandestinus,* four of the same aASVs were shared. This is out of a total of seven aASVs (57.1%) for *C. gayana* and 11 aASVs (36.4%) for *C. clandestinus*. *Paspalum* sp. and *C. gayana* share three bacterial aASVs out of 10 (30%) and seven (42.9%) aASVs, respectively. *Paspalum* sp. and *C. clandestinus* share three out of 10 (30%) aASVs from *Paspalum* sp. and 11 (27.3%) aASVs from *C. clandestinus*. There was one aASV being shared between all three genera, and this aASV is part of the family *Enterobacteriaceae.* There was no significant difference based on the Kruskal–Wallis test (Q > 0.05) within Shannon’s alpha diversity analysis between the different genera (Figure 2A). Figure 1B shows the aASVs from the cultivar Whittet of *C. clandestinus* from three different distributors within a cultivar., allowing a between seed distributors comparison. There are a total of 12 aASVs none of which are shared between all three distributors, with five different aASVs within the McKays seeds seed batch, three different aASVs within the Williams Group seed batch, and five different aASVs within the Anco seeds seed batch. The seed batch from Williams Group shares one out of its three aASVs (33.3%) with the seed batch from Anco seeds, which contained a total of five aASVs (20.0%). At the same time, the seed batch from McKays seeds shared no aASVs with either of the other two seed batches. There was a significant difference based on the Kruskal–Wallis test (Q < 0.05) within Shannon’s alpha diversity analysis between all three distributors (Figure 2B). Figure 1C shows the aASVs from different cultivars of *C. gayana* from a single distributor (Barenbrug), enabling a within-species, cultivar comparison, which only includes the bacterial communities of these four different cultivars. Within these seed batches, the cultivar Endura contained a total of three different aASVs, cv. Katambora contained a total of three different aASVs, cv. Mariner contained a total of two different aASVs, and cv. Tolgar contained a total of two different aASVs. Out of the total of three aASVs from cv. Endura, two were shared with three of the other cultivars (66.7%). Cv. Tolgar contains a total of two abundant aASVs and shares both of these with each of the three other cultivars (100.0%). The microbial community of cv. Katambora contains a total of three aASVs and shared two of those with each of the other three cultivars (66.7%). Cv. Mariner contains a total of two aASVs and shares both of these aASVs with all three other cultivars (100.0%). There were two aASVs that were shared between the four cultivars, which included the genera, *Pantoea* and *Pseudomonas*. There was no significant difference based on the Kruskal–Wallis test (Q > 0.05) within Shannon’s alpha diversity analysis between the cultivars Endura and Mariner, while all other cultivar comparisons showed a significant difference (Q < 0.05) (Figure 2C).

**Figure 3:**
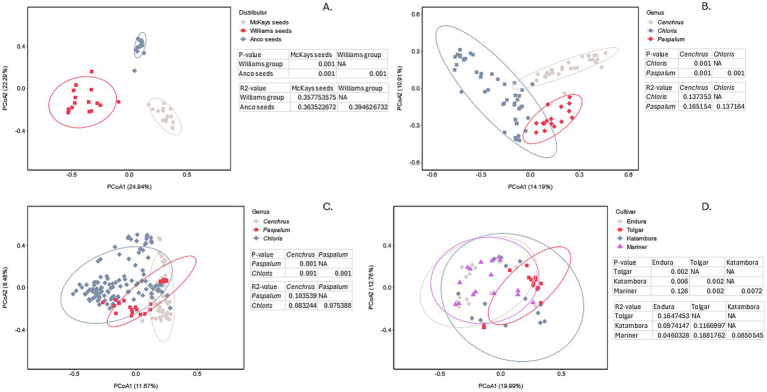
PCoA plot of bacterial communities including the PERMANOVA results showing the *p*-value indicating the significance of community differences and the R^2^ value representing the degree of separation between all parameters shown in the figures. **(A)** PCoA plot comparing the microbial communities of three different distributors of *Cenchrus clandestinus* from the cultivar Whittet. **(B)** PCoA plot comparing the microbial communities of the three different genera within the distributor Williams Group. **(C)** PCoA plot comparing the microbial communities of the three different genera within all distributors. **(D)** PCoA plot comparing the microbial communities of four different *Chloris gayana* cultivars from the distributor Barenbrug. Ellipse represented a 95% confidence interval.

To assess the variations of bacterial communities between different seed distributors, grass genera, and cultivars, permutational multivariate analysis of variance (PERMANOVA) and principal coordinates analysis (PCoA) were performed (Figure 3). Significant differences in microbial communities were represented by a *p*-value <0.05, and the degree of separation between communities was represented by R^2^ values. Looking at the population variance based on three different distributors of *C. clandestinus,* significant differences can be observed in cv. Whittet seed batches (*p* = 0.001, Figure 3A). The high R^2^ value indicates that the different distributors are a major contributing factor to the variation, which is also supported by the high percentage variance shown by PCoA1 and PCoA2. When comparing the microbial communities present in *Cenchrus, Chloris* and *Paspalum* within the distributor Williams Group (Figure 3B), it is shown that there is a significant difference between the three genera (p = 0.001). This is, however, accompanied by a relatively low R^2^ value (R^2^ < 0.2), suggesting that this comparison only explains a relatively small portion of the variation, which is also supported by relatively low PCoA1 and PCoA2 percentage variances. Similarly, Figure 3C shows significant differences (*p* = 0.001) between the three grass genera from all distributors. However, the low R^2^ values indicate that the microbial communities of the three grass genera could only explain a small proportion of the variance between the genera. This is also supported by the low percentage variance shown by PCoA1 and PCoA2. Looking at the PCoA plot showing variance between microbial communities present in four different cultivars of *C. gayana* from the distributor Barenbrug, significant differences (*p* < 0.05) are detected between all cultivars except for between Mariner and Endura (*p* = 0.126, Figure 3D). The R^2^ values being relatively low in contrast to the R^2^ values shown for the distributors indicates that the different cultivars only explain a small proportion of the variation between microbial communities. These results are supported by the percentages of PCoA1 and PCoA2 being in between the percentages of the genera and distributors.

#### Fungal communities

**Figure 4:**
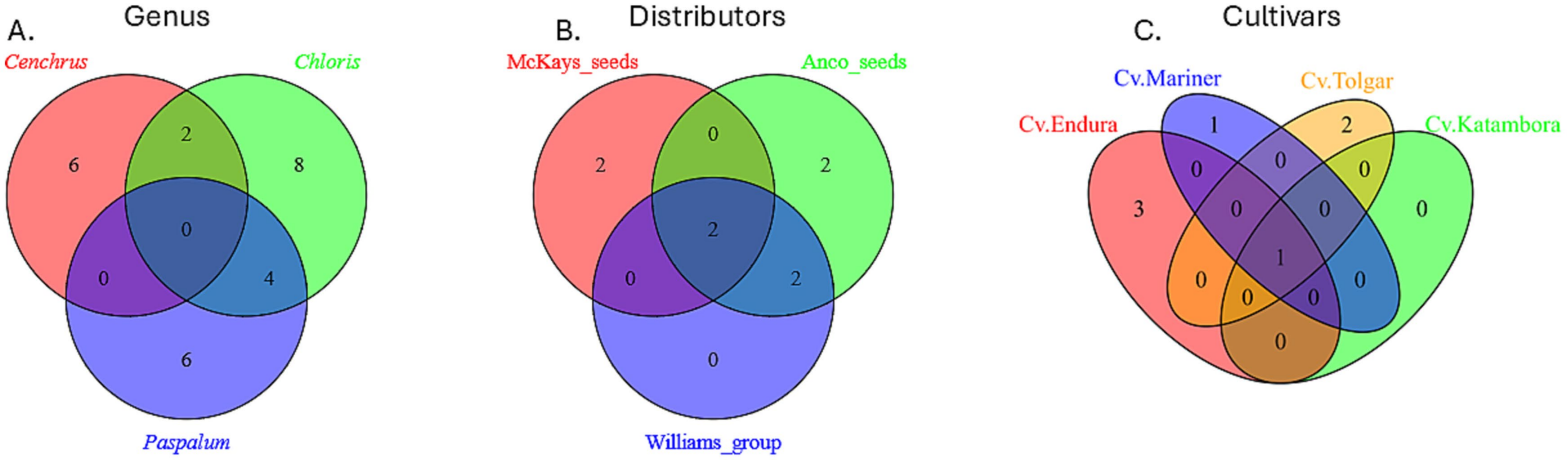
Venn diagram of fungal communities. **(A)** Comparing the abundant ASVs (aASVs) of three different genera. **(B)** Comparing the aASVs of three different distributors of *Cenchrus clandestinus* from the cultivar Whittet. **(C)** Comparing the aASVs of four different *Chloris gayana* cultivars from the distributor Barenbrug.

**Figure 5:**
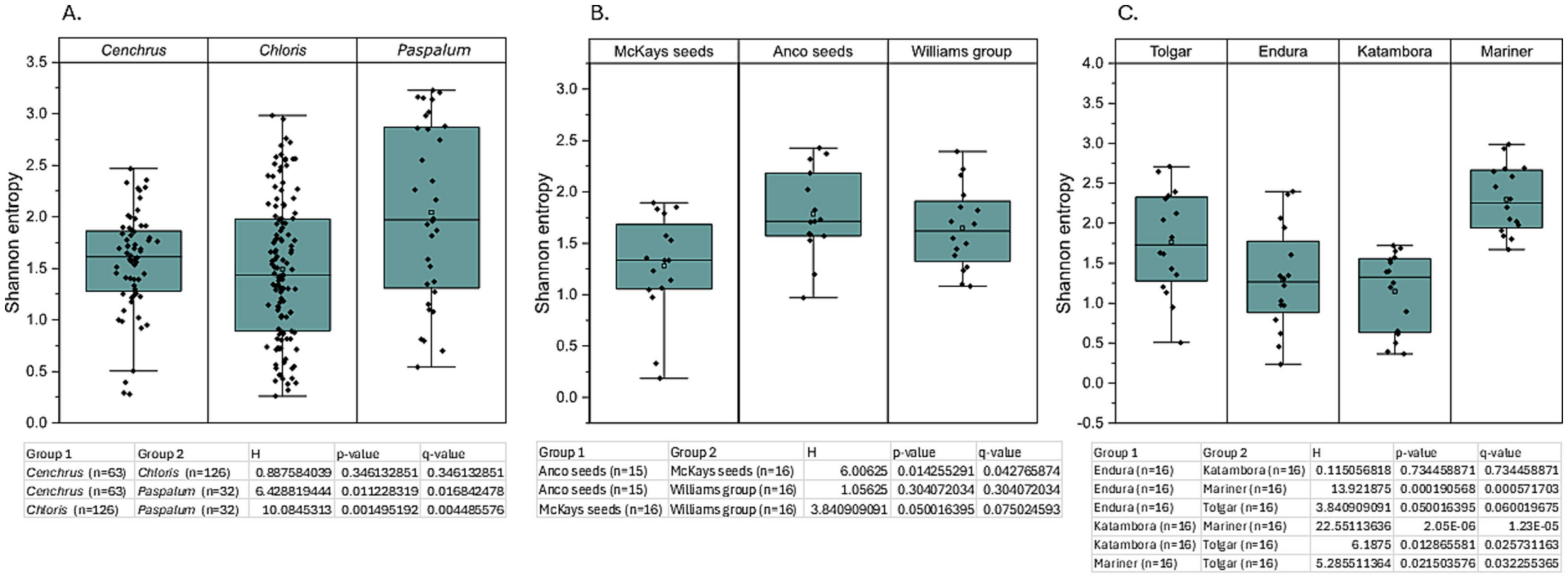
Shannon’s alpha diversity analysis based on pairwise Kruskal–Wallis test between fungal communities, including the H-value (Kruskal–Wallis test statistic), *p*-value (raw *p-*value from test), and Q-value [false discovery rate-corrected *p*-value (Benjamini–Hochberg correction)] comparing different parameters. **(A)** Shannon’s alpha diversity test comparing three different genera. **(B)** Shannon’s alpha diversity test comparing three different distributors of *Cenchrus clandestinus* from the cultivar Whittet. **(C)** Shannon’s alpha diversity test comparing four different *Chloris gayana* cultivars from the distributor Barenbrug.

The Venn diagram shows the fungal abundant ASVs (aASVs) on the genus level after combining all 16 replicates used for profiling. The ASVs with at least an abundance of 10% within the communities of the three genera (Figure 4A) show that from the eight abundant fungal ASVs present within the four *C. clandestinus* seed batches, two are shared with *C. gayana* (25.0%) and none are shared with *Paspalum* sp. The eight *C. gayana* seed batches have a total of 14 aASVs, of which two are shared with *C. clandestinus* (14.3%) and four are shared with *Paspalum* sp. (28.6%). The two *Paspalum* seed batches contain a total of 10 aASVs and share four with *C. gayana* (40.0%), while no aASVs were shared with *C. clandestinus.* Based on the Kruskal–Wallis test within Shannon’s alpha diversity analysis, there is a significant difference between the *Paspalum* and the other two genera (Q < 0.05), but no significant difference between *Cenchrus* and *Chloris* was observed (Q > 0.05) (Figure 5A). Figure 4B shows the fungal communities of *C. clandestinus* cv. Whittet seeds from three different distributors. This comparison only includes the communities present in these three seed batches. The seed batch from McKays seeds shares two out of four total aASVs (50.0%) with the seed batches from both Williams Group and Anco seeds. The community of the Williams Group shares two out of four aASVs with the McKays seeds batch (50.0%), and four out of four aASVs with the community present in the Anco seeds batch (100.0%). The Anco seeds batch shares two out of six fungal aASVs with the McKays seeds batch community (33.3%) and four with the Williams Group seed batch community (66.7%). These seed batches had two aASVs in common between them, which included *Sarocladium* and *Curvularia*. Based on the Kruskal–Wallis test within Shannon’s alpha diversity analysis, the seed batch from McKays seeds is significantly different from the Anco seeds seed batches (Q < 0.05), while there is no significant difference between the Williams Group and the other two seed batches (Q > 0.05) (Figure 5B). The comparison, which only includes the communities present in four different cultivars of *C. gayana* from the distributor Barenbrug (Figure 4C), shows that cv. Endura has four aASVs and shares one single aASV with all three of the other cultivars (25.0%). Cv. Tolgar contains three aASVs and shares one aASV with all three of the other cultivars (33.3%). The cultivar Katambora has one aASV and shares this aASV with all three other cultivars (100.0%). Finally, cv. Mariner contains two aASVs and shares one of these aASVs with the other three cultivars (50.0%). These four seed batches from four cultivars only shared one common aASV from the family *Didymellaceae*. Based on the Kruskal–Wallis test within Shannon’s alpha diversity analysis shown in Figure 5C, there is no significant difference between the cultivar Endura and the cultivars Katambora and Tolgar (Q > 0.05), while there is a significant difference between all other fungal communities present within the different cultivars (Q < 0.05).

**Figure 6:**
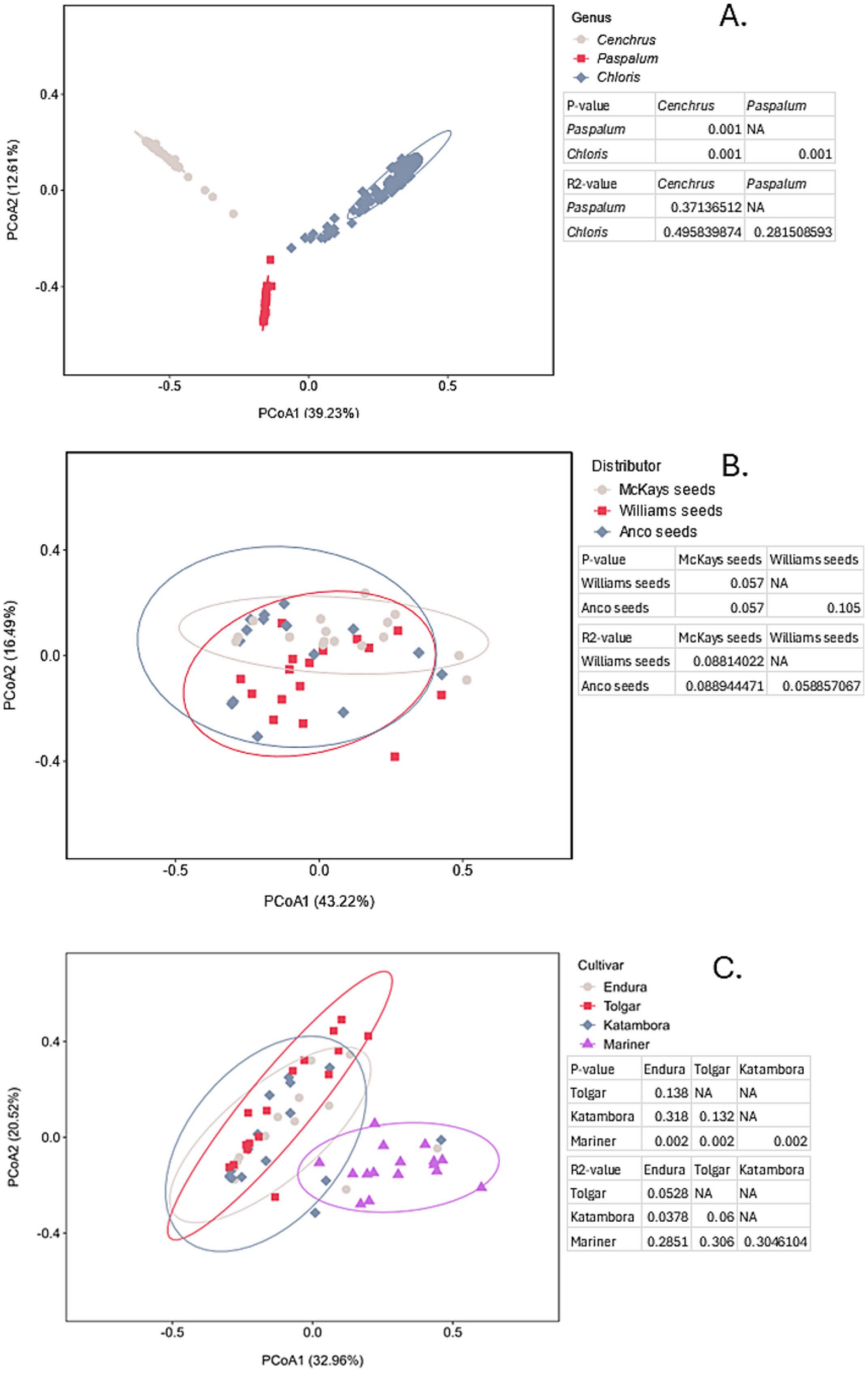
PCoA plot of fungal communities, including the PERMANOVA results, showing the *p*-value indicating the significance of community differences and the R^2^ value representing the degree of separation between all parameters, shown in the figures. **(A)** PCoA plot comparing the microbial communities of the three different genera within all distributors. **(B)** PCoA plot comparing the microbial communities of three different distributors of *Cenchrus clandestinus* from the cultivar Whittet. **(C)** PCoA plot comparing the microbial communities of four different *Chloris gayana* cultivars from the distributor Barenbrug. Ellipse represented a 95% confidence interval.

To assess the variations of fungal communities between different grass genera, seed distributors, and cultivars, permutational multivariate analysis of variance (PERMANOVA) and principal coordinates analysis (PCoA) were performed (Figure 6). The PCoA plot comparing the three grass genera indicates there is a significant difference (*p* = 0.001) between the fungal communities of the three genera (Figure 6A). This is supported by the fact that the R^2^ values are quite high between the communities, which indicates that grass genera are a major contributing factor to the microbial differences of the seeds. Looking at the variation between *C. clandestinus* cv. Whittet communities of three different distributors, the *p*-values show that the different distributors are not significantly different (*p* > 0.05) from one another (Figure 6B). This is again apparent in the R^2^-values, with all of the values being between 0.06 and 0.09. The PCoA1 and PCoA2 percentages also support this claim. A PCoA plot showing the variation between four different cultivars of *C. gayana* indicates that there is a significant difference (*p* < 0.05) between mariner and the other three cultivars, while there is no significant difference between the cultivars Endura, Tolgar, and Katambora (Figure 6C). This is again supported by the R^2^ values, as the R^2^ values between Endura, Katambora, and Tolgar are very low, while those between Mariner and the other three show clear variation.

### Microbial profile of warm-season grasses

#### Bacterial profile

**Figure 7:**
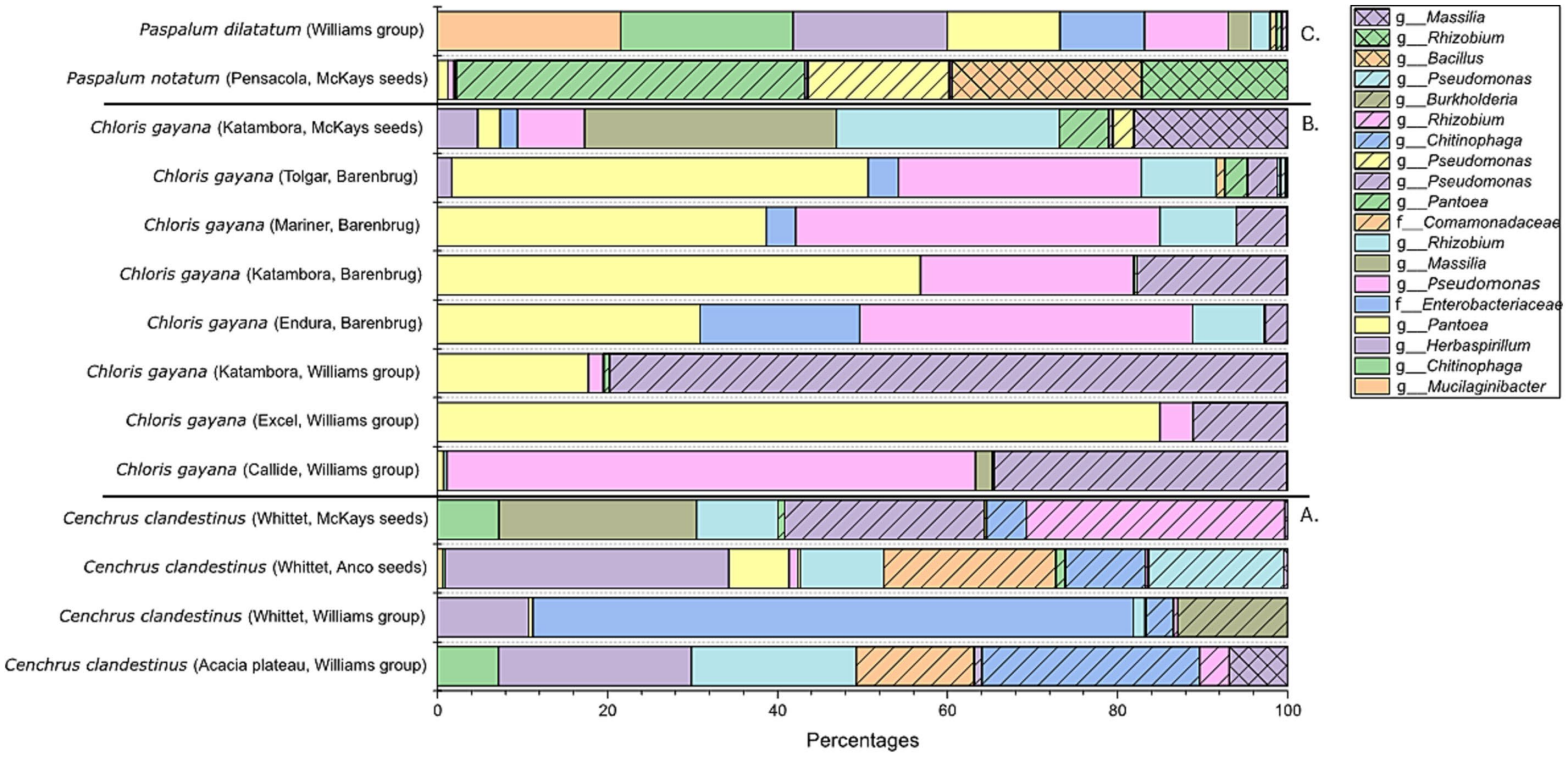
Bacterial microbiome profile of all 14 seed batches from the three genera of warm-season pasture grasses. This is shown in a 100% stacked bar plot, which only includes ASVs with an abundance higher than 10%. **(A)** Contains the bacterial profiles of the *Cenchrus clandestinus* seed batches. **(B)** Contains the bacterial profiles of the *Chloris gayana* seed batches. **(C)** Contains the bacterial profiles of the *Paspalum* sp. seed batches.

The bacterial profiles after combining the 16 replicates of each seed batch were further compared based on aASVs and shown in a 100% bar chart (Figure 7). The seed microbiome present in the four *C. clandestinus* seed batches showed clear differences between each other (Figure 7A). Cv. Acacia plateau from the Williams group contains aASVs from the genera *Chitinophaga*, *Rhizobium,* and *Herbaspirilium* and an ASV from the family *Comamonadaceae*. Cv. Whittet from the Williams Group contains aASVs from the genera *Herbaspirilium* and *Burkholderia* and an ASV of the *Enterobacteriaceae* family. Cv. Whittet from Anco seeds contains aASVs from the genera *Herbaspirilium*, *Rhizobium,* and *Pseudomonas* and an ASV from the family *Comamonadaceae*. Cv. Whittet from McKays seeds group contains bacterial aASVs from the genera *Rhizobium, Pseudomona,* and *Massilia*. In contrast to the *C. clandestinus* seed batches, looking at all eight seed batches from *C. gayana* (Figure 7B), there are three ASVs that are highly abundant in the majority of the *C. gayana* seed batches. These three ASVs include two *Pseudomonas* species and an ASV of the genus *Pantoea*. The only outlier is the seed batch of cv. Katambora from the distributor McKays seeds. This seed batch contains one of the two *Pseudomonas* species, as well as aASVs of the genera *Massilia* and *Rhizobium*. The *Paspalum notatum* seed batch contains aASVs of the genera *Pantoea*, *Pseudomonas*, *Bacillus,* and *Rhizobium*, while the *Paspalum dilatatum* seed batch contained aASVs of the genera *Mucilaginibacter*, *Chitinophaga*, *Herbaspirillum,* and *Pantoea* (Figure 7C).

#### Fungal community

**Figure 8:**
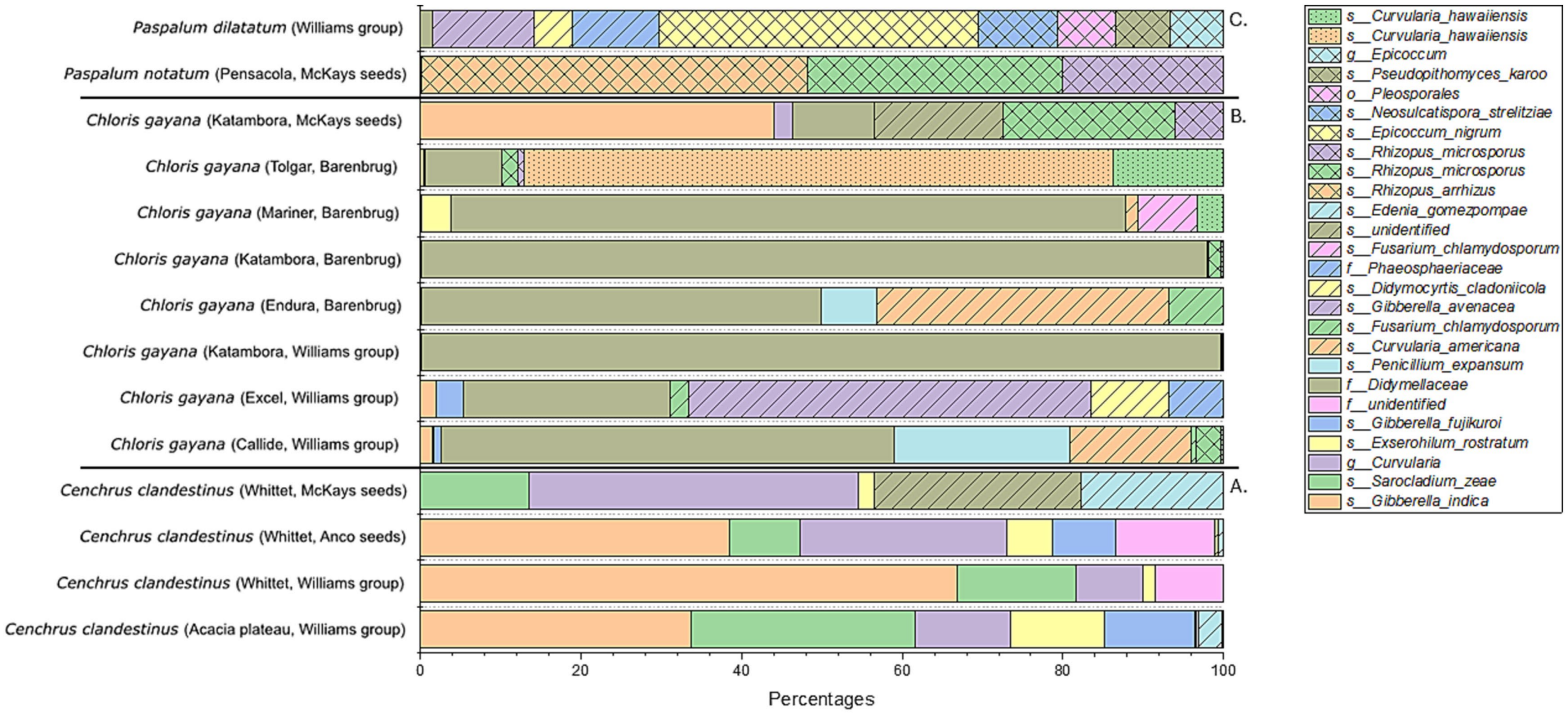
Fungal microbiome profile of all 14 seed batches from the three genera of warm-season pasture grasses. Shown in a 100% stacked bar plot which only includes ASVs with an abundance higher than 10%. **(A)** Contains the fungal profiles of the *Cenchrus clandestinus* seed batches. **(B)** Contains the fungal profiles of the *Chloris gayana* seed batches. **(C)** Contains the fungal profiles of the *Paspalum* sp. seed batches.

The fungal profiles after combining the 16 replicates of each seed batch were further compared based on aASVs and shown in a 100% bar chart (Figure 8). For the four *C. clandestinus* seed batches, it can be seen that *Curvularia* and *Sarocladium* species are present in all four seed batches (Figure 8A). Three seed batches contain *Gibberella* sp. (cv. Acacia plateau from Williams Group, cv. Whittet from Williams group, and cv. Whittet from Anco seeds), two contain a different *Gibberella* sp. (cv. Acacia plateau from Williams Group and cv. Whittet from Anco seeds), one contains an *Exserohilum* sp. (cv. Acacia plateau from Williams Group), and one seed batch (cv. Whittet from McKays seeds) contains an abundant *Edenia* sp. and an unidentified ASV. Overall, eight seed batches from *C. gayana* (Figure 8B) contain one *Didymellaceae* family ASV that is highly abundant in the majority of the *C. gayana* seed batches. However, there are four other ASVs that are highly abundant within some of the seed batches, and these include aASVs from the genera *Pennicillium* (cv. Callide from the Williams Group), *Curvularia* (cv. Callide from the Williams Group and cv. Endura from Barenbrug), *Gibberella* (cv. Excel from the Williams Group), and two different *Curvularia* ASVs (cv. Tolgar from Barenbrug). The final seed batch cv. Katambora from McKays seeds contains three completely different aASVs. These include ASVs from the genera *Gibberella* and *Rhizopus* and an unidentified fungal microbe. The abundant ASVs present in the microbial communities for the *Paspalum* species include the different species within the genus *Rhizopus* for *Paspalum notatum* cv. Pensacola from the distributor McKays seeds and two aASVs of the genera *Epicoccum* and *Gibberella* for *Paspalum dilatatum* from the distributor Williams Group (Figure 8C).

### Microbial culturability

#### Bacterial culturability

**Figure 9:**
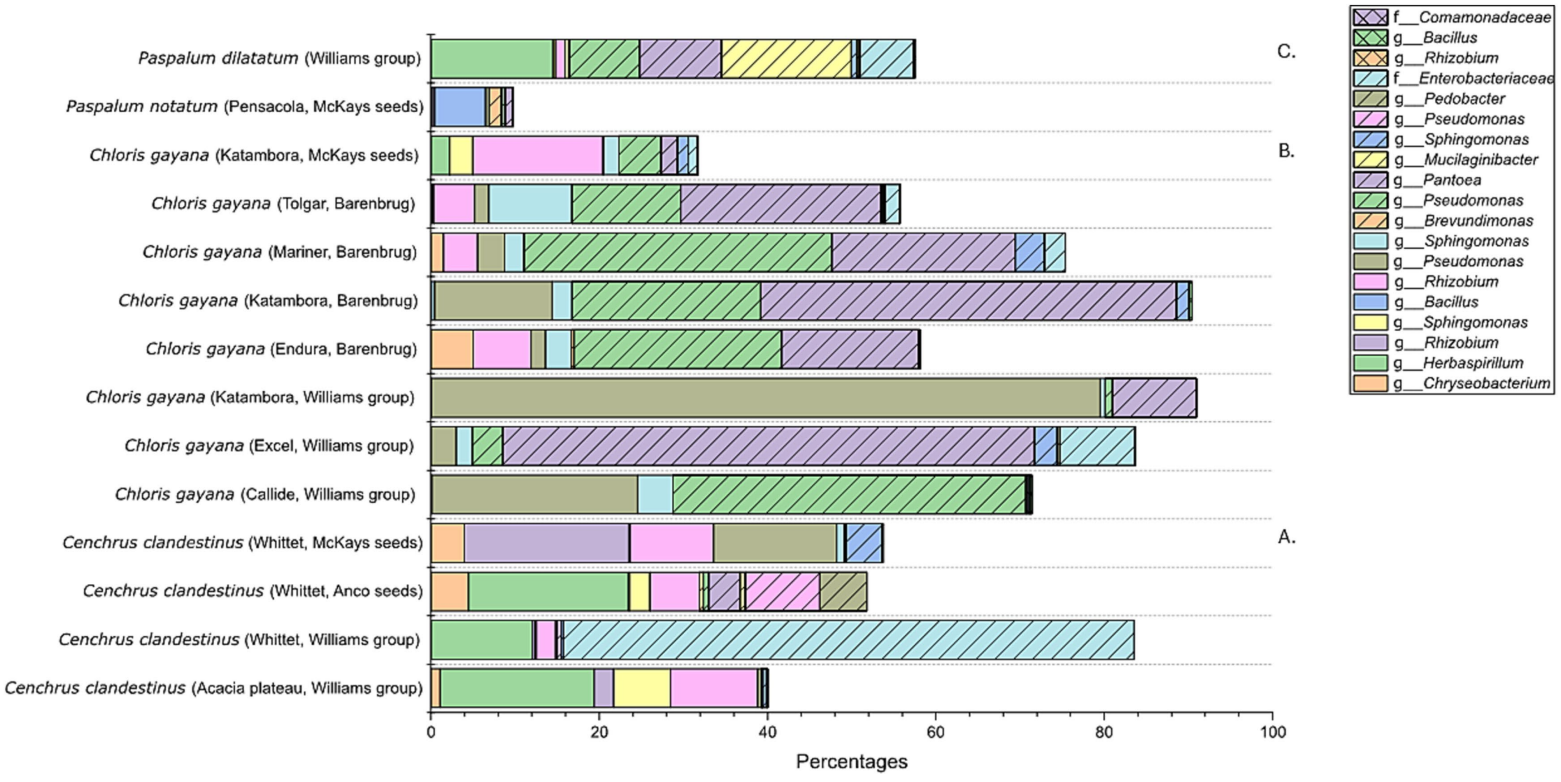
Percentages of culturable bacteria within the 16S profile. **(A)** The percentages of culturable bacteria within the four *Cenchrus clandestinus* seed batches. **(B)** The percentages of culturable bacteria within the eight *Chloris gayana* seed batches. **(C)** The percentages of culturable bacteria within the two *Paspalum* sp. seed batches.

For each of the 14 warm-season grass seed batches, five replicates were combined for the isolation with a total of 95 bacteria which were isolated and analysed. The 16S sequences of all isolates were compared to identify corresponding ASVs in the 16S amplicon data. Figure 9 shows the percentage of the profiles from the different seed batches that match culturable bacteria. In general, *C. gayana* seed microbes were more culturable in our experimental setup (32–91%) compared to a range of 40–84% for *C. clandestinus* and a range of 10–58% for the *Paspalum* sp. Figure 9A shows the percentages of culturable bacterial microbes present in the profiles of the four seed batches from *C. clandestinus.* Looking at cv. Acacia plateau from the distributor Williams Group, 40% of the ASVs from the profile came from culturable bacteria. For the seed batch cv. Whittet from the distributor Williams Group, 84% of the ASVs from the profile were culturable bacteria. The seed batch cv. Whittet from the distributor Anco seeds shows that 52% of the ASVs from the profile came from culturable bacteria. Cv. Whittet from the distributor McKays seeds showed 53% of the ASVs from the profile consisted of culturable bacteria. Figure 9B shows the percentages of culturable bacterial microbes present in the profiles of the eight seed batches from *C. gayana.* The cv. Callide from the Williams Group showed that 71% of the profile consisted of culturable bacteria. For cv. Excel from the distributor Williams Group, 84% of the profile consists of culturable bacteria. The seed batch cv. Katambora from the distributor Williams Group shows that of the profile, 91% of the reads were from culturable ASVs. For the cv. Endura from the distributor Barenbrug, it can be seen that within the profile, 58% were from culturable bacteria. Cv. Katambora from Barenbrug shows that 90% of the profile were from culturable bacteria. *C. gayana* cv. Mariner from the distributor Barenbrug showed that 75% of the profile consists out of culturable ASVs. For the seed batch cv. Tolgar from the distributor Barenbrug, of the total reads from the profile, 56% were from culturable. The *C. gayana* seed batch is cv. Katambora from the distributor McKays seeds and shows that 32% of the total profile consists of culturable bacteria. Looking at the seed batches from two *Paspalum* species (Figure 9C), it can be seen that for *Paspalum notatum,* only 10% of the profile consists of culturable ASVs. For *Paspalum dilatatum*, 58% of the reads from the profile came from culturable ASVs. Overall, from all 14 seed batches, the culturable ASVs came from 10 highly abundant bacteria from the genera, *Herbaspirillum, Rhizobium, Pseudomonas, Pantoea, Sphingomonas,* and *Mucilaginibacter*, and the family *Enterobacteriaceae.*

#### Fungal culturability

**Figure 10:**
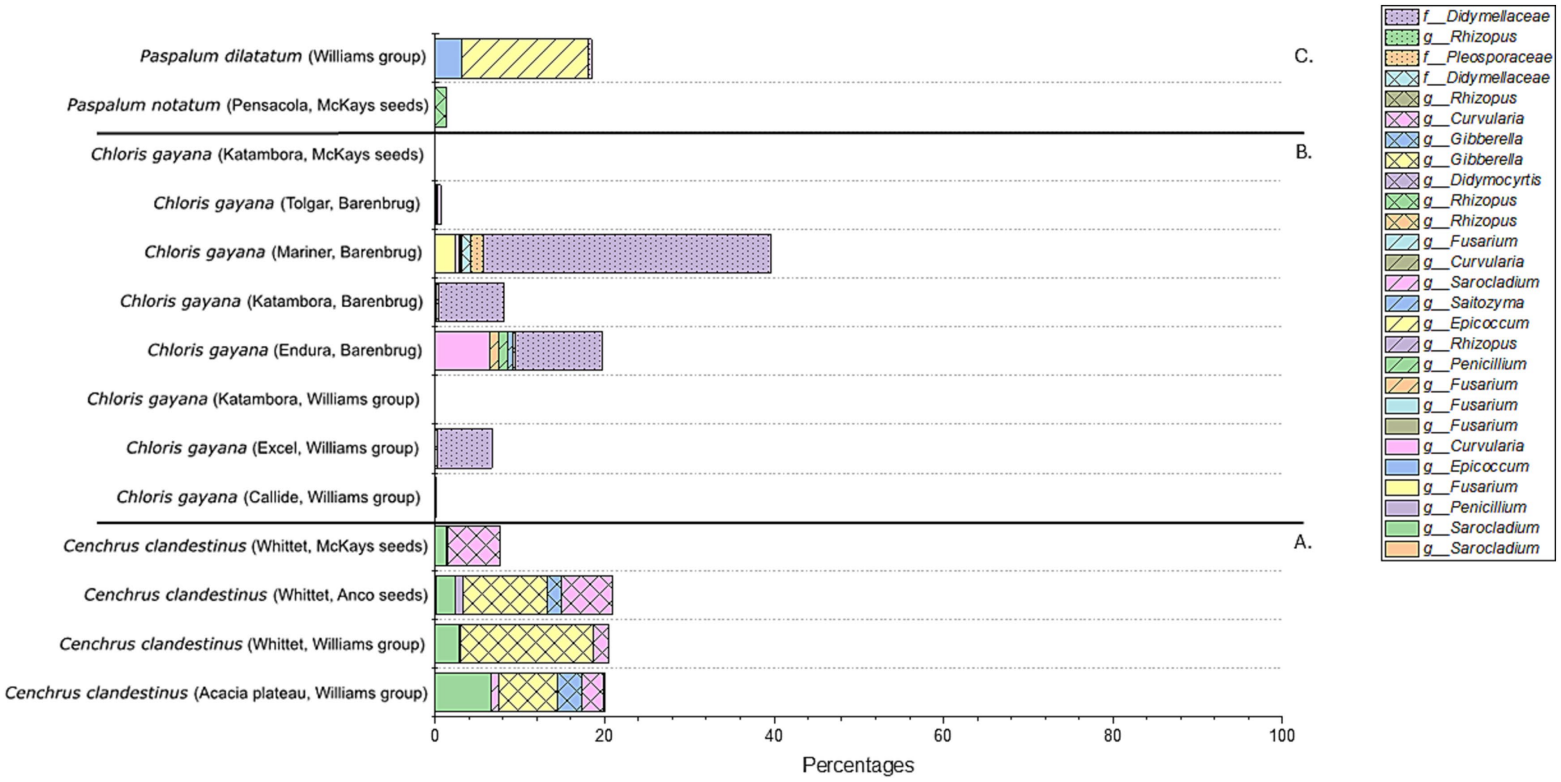
Percentages of culturable fungi within the ITS profile. **(A)** The percentages of culturable fungi within the four *Cenchrus clandestinus* seed batches. **(B)** The percentages of culturable fungi within the eight *Chloris gayana* seed batches. **(C)** The percentages of culturable fungi within the two *Paspalum* sp. seed batches.

For each of the 14 warm-season grass seed batches, five replicates were combined for isolation, with 37 fungi that were isolated and analysed. For this analysis, the ITS sequences of all isolates were used to find the corresponding ASVs. Figure 10 shows the abundance of the isolates within the profiles of the different seed batches. Overall, it can be seen that the fungal profiles contain a significantly smaller percentage of culturable microbes than the bacterial profiles. From the *C. clandestinus* seed batches, only between 8 and 21% of the total profiles come from culturable fungi. For the *C. gayana* seed batches, the culturability ranges between 0 and 39%, and for the two *Paspalum* species, this range was between 2 and 18%. Figure 10A shows the percentages of culturable fungal microbes present in the profiles of the four seed batches from *C. clandestinus.* In cv. Acacia plateau from the distributor Williams Group, 20% of the ASVs from the profile came from culturable fungi. For the seed batch cv. Whittet from the distributor Williams Group, 20% of the ASVs from the profile were culturable fungi. The seed batch cv. Whittet from the distributor Anco seeds shows that 21% of the ASVs from the profile came from culturable fungi. Cv. Whittet from the distributor McKays seeds showed only 8% of the ASVs from the profile consisted of culturable fungi. Figure 10B shows the percentages of culturable fungal microbes present in the profiles of the eight seed batches from *C. gayana.* For these seed batches, it can be seen that within the profiles of cv. Callide from the Williams Group, cv. Katambora from Williams group, cv. Tolgar from Barenbrug, and cv. Katambora from McKays seeds, only 0–1% is from culturable fungi. The four outliers are cv. Excel from Williams Group, which shows that 7% of the profile comes from culturable fungi. Cv. Endura from Barenbrug, which shows 19% of the profile is from culturable fungi. Cv. Katambora from the distributor Barenbrug shows that 8% of the fungal profile comes from culturable fungi. Cv. Mariner from Barenbrug shows that 39% of the profile consists of culturable fungi. Looking at the seed batches from two *Paspalum* species (Figure 10C), it can be seen that for *Paspalum notatum,* only 2% of the profile consists of culturable ASVs. For *Paspalum dilatatum*, 18% of the reads from the profile came from culturable ASVs. The culturable ASVs from all seed batches were largely part of the genera *Gibberella, Curvularia,* and *Epicoccum* and the family *Didymellaceae*.

## Discussion

The aim of this study was to understand the bacterial and fungal communities in warm-season pasture grass seeds and what influences these communities and the culturability of these microbes. This would give a clear insight into what microbes are present in these communities and can give an indication on how they can be utilised for agronomic purposes (Chesneau et al., 2022). Plant bacterial and fungal communities have been reported to change based on different parameters in plants (Brigham et al., 2023). In addition, in a creeper of the mint family, *Glechoma hederacea,* it has been shown that a significant portion of both the bacterial and fungal microbiome is vertically transferred to the next generation (Vannier et al., 2018). This is also shown in the seeds of a range of different plants, including herbs, vines and trees, where it has been shown that both bacterial and fungal communities are driven by the host genetics (Liu et al., 2022). While in another study, the bacterial and fungal microbiota were shown to be harboured within different rice seed compartments, which could result in differences in drivers between the bacterial and fungal communities (Eyre et al., 2019). Bacterial and fungal communities behaving differently has been shown in a range of different plant, soil, and seed samples before (Lepinay et al., 2024; Gan et al., 2022; Kim and Lee, 2021). However, understanding how these differences can be exploited in warm-season pasture grasses could be very beneficial for the future of the dairy industry in temperate climates.

### Distributor effect on bacterial communities

The results of this study have shown that *C. clandestinus* cv. Whittet from three distributors contain significantly different bacterial communities with high R^2^ values, indicating a strong distributor effect. Two possible reasons for a ‘distributor’ effect could be the geographic location of production and/or the methodologies of processing seeds by the distributors, including storage practices, harvesting practices, and post-harvest treatments. The geographic location could play a role due to the fact that the grasses used for seed production were grown under different climatic conditions and/or in different soil types, which promotes the abundance of certain microbes while harbouring a core microbial community (Tabassum et al., 2024). The three different distributors from which the seeds in this study were sourced mostly produced seeds in the same region around the New South Wales (NSW) and Queensland border in Australia. This indicates that most seeds were produced in similar climatic conditions. The soil type of these production fields, however, could still be a contributing factor to the difference in the bacterial communities within plant seeds (Nelson, 2018). A study on perennial ryegrass has shown that there were significant differences in the bacterial communities when seeds were grown within the same approximate area with different soil types within New Zealand (Tannenbaum et al., 2021). This could potentially be the same in warm-season pasture grasses, suggesting that the soil type might still be a driver of the ‘distributor’ differences. The storage of seeds has also been shown to have an impact on the bacterial communities of *Glycine max* and *Acacia ulicifolia* seeds (Chandel et al., 2021; Russell et al., 2025). This indicates that the microbiome differences between seed batches observed in our study could also be a result of varying storage practices used by different distributors. Another post-harvest treatment effect is seed coating, which is applied to the seeds. The effects of seed coating has been observed in a study on soybean where the microbial communities were especially affected by the inclusion of biocontrol agents in the seed coating (Yang et al., 2023; Kim and Lee, 2021). Furthermore, most of the abundant bacterial microbes of warm-season grass seeds could possibly be located around the seed coat instead of within the endosperm or embryo of seeds, as has been shown in a study in wheat (Berg and Raaijmakers, 2017; Kuźniar et al., 2020). This would make the bacterial microbiome very susceptible to seed processing methods employed by the different distributors. Visual differences between seed batches were observed in this study (Supplementary Figure 2), as some different seed batches had visually different seed coatings. The effects of such coatings on warm-season grass seed microbiomes will need to be assessed. Collaborations with distributors will be required to further explore these variables while maintaining commercial sensitivities around proprietary coatings.

### Host effect on bacterial communities

This study also shows that the bacterial communities present within the *Cenchrus, Chloris,* and *Paspalum* seed batches from the distributor Williams Group are significantly different from one another. However, the R^2^ values are much lower compared to what can be seen in the ‘distributor’ comparison, indicating that in commercially available warm-season pasture grass seeds, the host genetics do not drive the bacterial communities as much as the ‘distributor’ does. A similar trend is observed when focussing on the bacterial communities present within seed batches of three different genera but from the same distributor. The R^2^ values are shown to be higher within this comparison, indicating that the difference in microbial communities based on the host genetics is less prominent when all 14 seed batches are included. This could possibly be a result of many more variables being included when comparing bacterial communities present within the genera, including all 14 seed batches, which could affect separation (Shi et al., 2020). In summary, the results do indicate that the hosts drive the bacterial communities within warm-season pasture grasses. However, the fact that the seed processing by different distributors is still a stronger driver of the bacterial communities indicates opportunities to adjust the bacterial communities to aid plant growth even though there is a core microbiome driven by the host genetics (Hadian et al., 2025; Guha and Mandal Biswas, 2024). The modification of bacterial communities to aid plant growth would need to be investigated further.

### Host effect on fungal communities

The results from examining the fungal communities have shown that, unlike bacterial communities, the differences in the fungal communities present in warm-season pasture grasses are mainly driven by the host. The largest difference is observed when comparing the *Cenchrus, Chloris,* and *Paspalum* seed batches from all distributors. This was also shown in a study looking at the fungal communities within *Glechoma hederacea,* which showed that plants transfer some core fungal microbes vertically through generations (Vannier et al., 2018). This study on the effects of host genetics on fungal communities showed that there is a selective stage of plants where fungal microbes are being filtered before being transferred to the next generation. In a specific example, endophytic fungi *Epichloë festucae* form asymptomatic infections within cool-season grass stems and seeds, and these endophytic fungi are transferred to the next generation (Clay and Schardl, 2002). This process could be very similar for any endophytic fungi that might be present in the warm-season grass seeds, but this hypothesis needs to be investigated further to understand this process. The fact that fungal microbes seem to be selected based on host genetics more than through the processing of the seeds could give an advantage in predicting the fungal communities present in seeds of the genera when the core fungal microbes are highlighted (Malacrinò et al., 2023). However, in contrast to the bacterial communities, the fungal communities seem to have a much stronger core fungal community with much less microbial variation between seed batches. This might result in the seed fungal microbiome being more difficult to adjust with different species or genera of beneficial fungal microbes to aid in growth under different conditions. However, there are opportunities to enhance growth by inoculating seeds with different strains of core fungal microbes (Luo and Tian, 2021). Any possibilities of altering the core fungal communities would need to be investigated further.

### Culturability of bacterial communities

The culturability results have shown that between 40 and 84% of the *C. clandestinus* seed batch profiles came from culturable bacteria, while between the 32 and 91% of the *C. gayana* profiles came from culturable bacteria and between 10 and 58% for the *Paspalum* species profiles came from culturable bacteria. A previous study has shown that in temperate grasses, most of the bacteria from the profile were culturable, with 18 out of the 24 genera (75%) being present in the temperate grass profile (Tannenbaum et al., 2020). Our study has shown that the culturability of the bacterial microbiome of warm-season grass seeds and temperate grass seeds are relatively similar, with taxa both abundant and rare in the 16S profile being culturable. The aASVs, which were not culturable within our study came from three genera and one family. The three missing ASVs included the genera *Chitinophaga, Massilia,* and *Burkholderia,* which were found not to grow optimally under the conditions described in this study (Yao et al., 2021; Altankhuu and Kim, 2017; Zhang et al., 2006; Marrs et al., 2021). The results described in this study also show that, on average, 60% of the aASVs from the bacterial profile were culturable. These culturable aASVs were from the genera *Herbaspirillum, Rhizobium, Sphingomonas, Pseudomonas, Pantoea,* and *Mucilaginibacter.* All six of these genera have shown to contain plant growth-promoting or bio-protection capabilities (Wiggins et al., 2022; Gen-Jiménez et al., 2023; Chakraborty et al., 2021; Asaf et al., 2018; Berendsen et al., 2015; Sarkar et al., 2022; Chen et al., 2017; Fan and Smith, 2022). With a total of 96 isolates that were obtained from warm-season pasture grasses, the previously named isolates, along with all the other lower abundant isolates, will need to be investigated further. This investigation would include characterising isolates for possible plant growth-promoting features under different conditions, including biotic and abiotic stresses.

### Culturability of fungal communities

In our study on fungal communities of the 14 warm-season pasture grass seed batches, only between 1 and 39% of the fungal profiles came from culturable microbes under the conditions used. At the same time, a previous study has shown that isolating fungal microbes is an effective way of analysing the microbial community present in root, shoot, and soil samples (Owen and Hundley, 2004). This seems not to be the case for the seed fungal microbes within warm-season pasture grasses. The aASVs within the 14 profiles which were not culturable include the genera *Exserohilum, Edenia, Didymocyrtis,* and *Pseudopithomyces*. In general, fungi are more difficult to isolate than bacteria and have more specific requirements for growth. For the genera *Exserohilum, Edenia* and *Pseudopithomyces* studies have shown that these fungi grow better under conditions with higher temperatures and higher humidity (Da Cunha et al., 2012; Del Serrone and Fornasari, 1995; Gonzalez et al., 2007; Collin and Towers, 1995). At the same time, the genus *Didymocyrtis* is shown to not perform well on PDA media and would need to be grown on *Dothistroma* medium instead to have better growth (Monteiro et al., 2022; Gwon et al., 2021). To try to culture every single ASV from a profile, these different conditions and medias need to be utilised to make sure the optimal conditions are met for each of the abundant ASVs. Different isolation protocols have been explored, which could be utilised to increase the culturability of some fungal microbes (Li et al., 2023). Looking at the isolated fungal genera within this study, there are isolates from the genera *Sarocladium, Gibberella, Curvularia,* and *Epicoccum*. All four of these isolated fungal microbes have been shown to contain bioprotectant or biostimulant functions (Błaszczyk et al., 2021; Keswani et al., 2022; Redman et al., 2011; Nzabanita et al., 2024). With a total of 37 isolates obtained from warm-season pasture grasses, the previously named isolates along with all the other lower abundant isolates will need to be investigated further. This includes characterising isolates to possibly aid plant growth under different conditions, including both biotic and abiotic stresses.

## Conclusion

The aim of this study was to examine and explore differences in bacterial and fungal microbial communities present in commercially available warm-season pasture grasses and create a library of possible beneficial microbes for further exploration. The results described in this research show that bacterial communities are likely easily affected by the processing of seeds by different distributors. This suggests that these communities are variable and more difficult to predict, but it also indicates that these bacterial communities could be adjusted to create a more beneficial bacterial microbiome for pasture establishment and growth. Fungal communities are mainly affected by the genetics of the host grasses, and this suggests that these communities are a lot more predictable between host species. This could, however, mean that adjusting these communities with different species would be more challenging as they are less variable. However, the possibility of enhancing growth through inoculating seeds with different strains of core fungal microbes could be explored. Most of the highly abundant bacterial microbes are culturable from the grass seeds with, on average, approximately 60% of the bacterial profiles being culturable, resulting in a pool of bacteria being available for functional studies. While for the fungal profiles, only approximately 12% of the abundant microbes came from culturable ASVs, implying more isolation work needs to be done to open up warm-season grass seed associated fungi for further studies.

## Data Availability

The names of the repository/repositories and accession number(s) can be found at: https://www.ncbi.nlm.nih.gov/, PRJNA1249115.
