# Supplementary figures and images for: The diversity, dynamics, and culturability of bacterial and fungal communities present in warm-season pasture grass seeds
Source: Front Microbiol. 2025 Jun 25;16:1621463. doi: 10.3389/fmicb.2025.1621463 (PMC12237998; doi:10.3389/fmicb.2025.1621463)

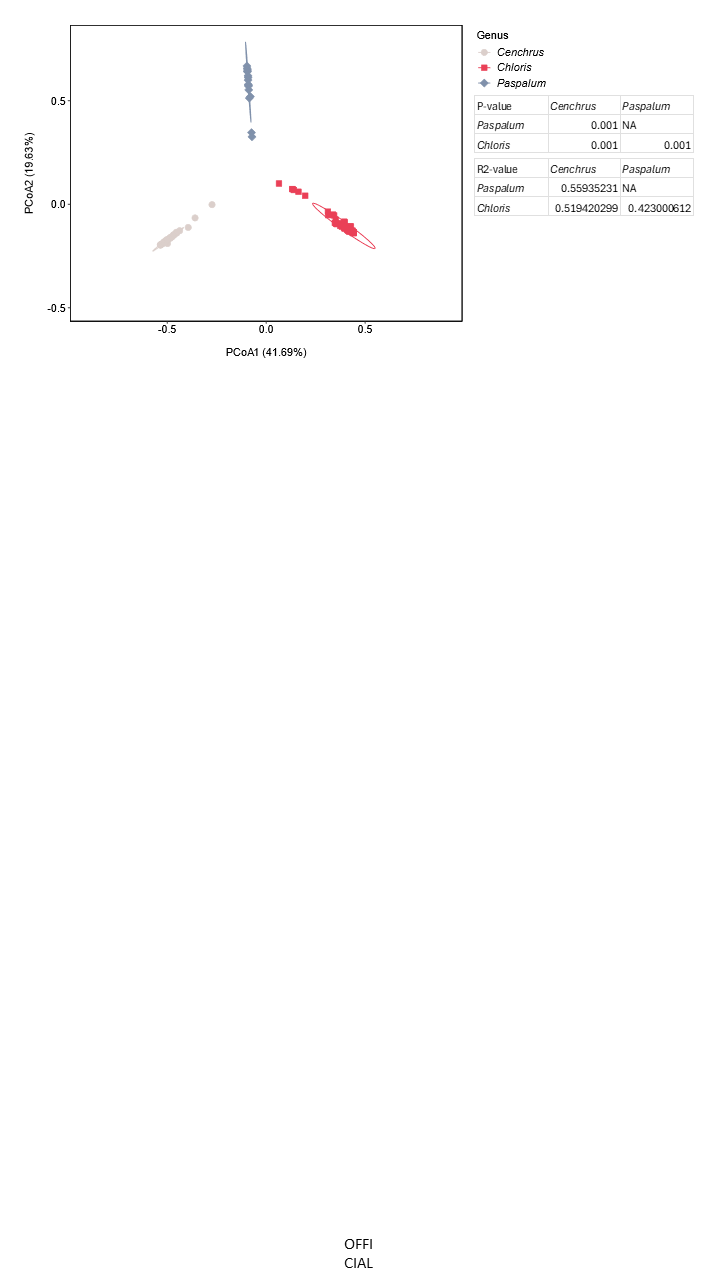

Supplement: Supplementary file 1 [file Image_1.tif]

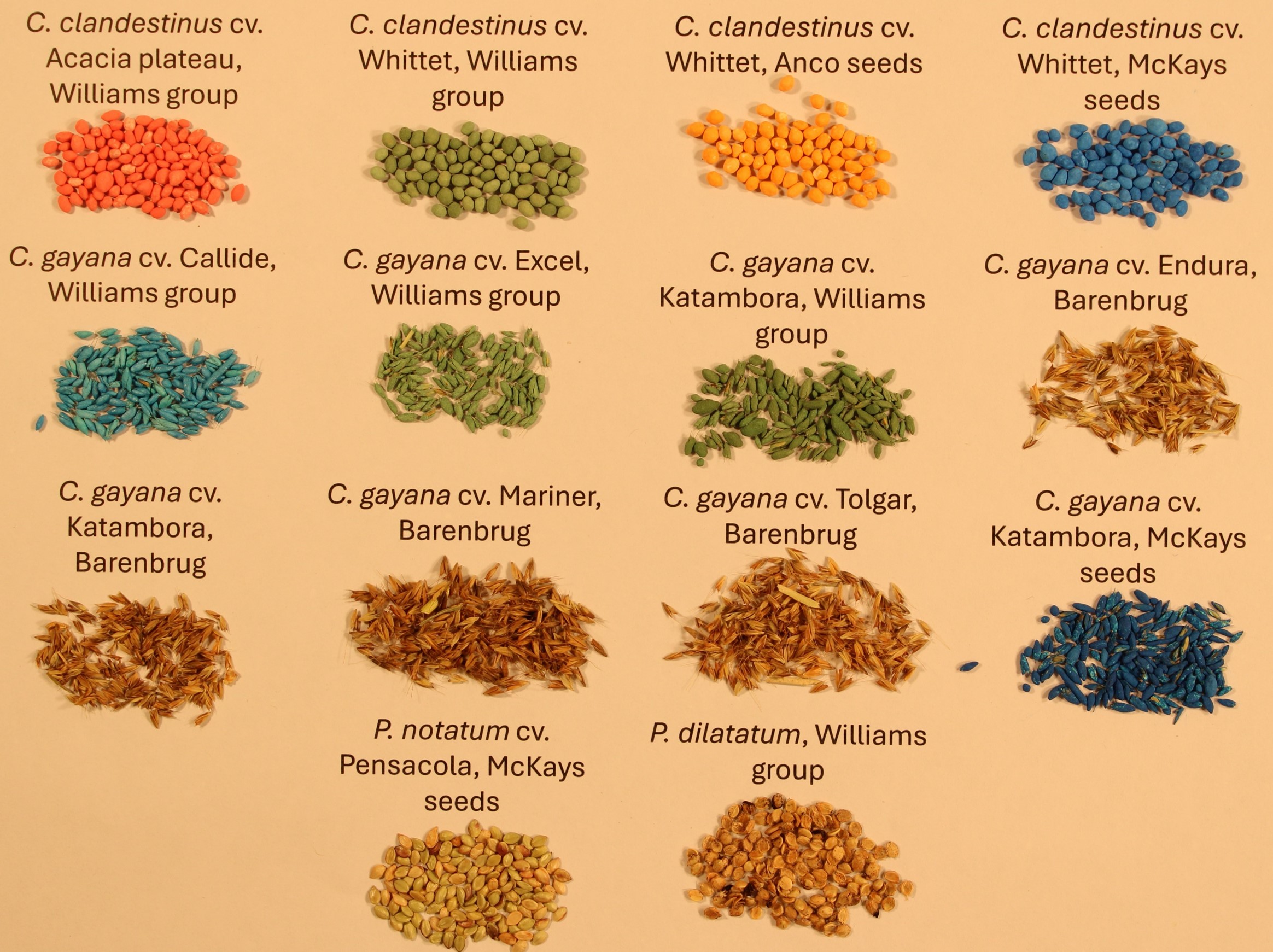

Supplement: Supplementary file 2 [file Image_2.jpg]

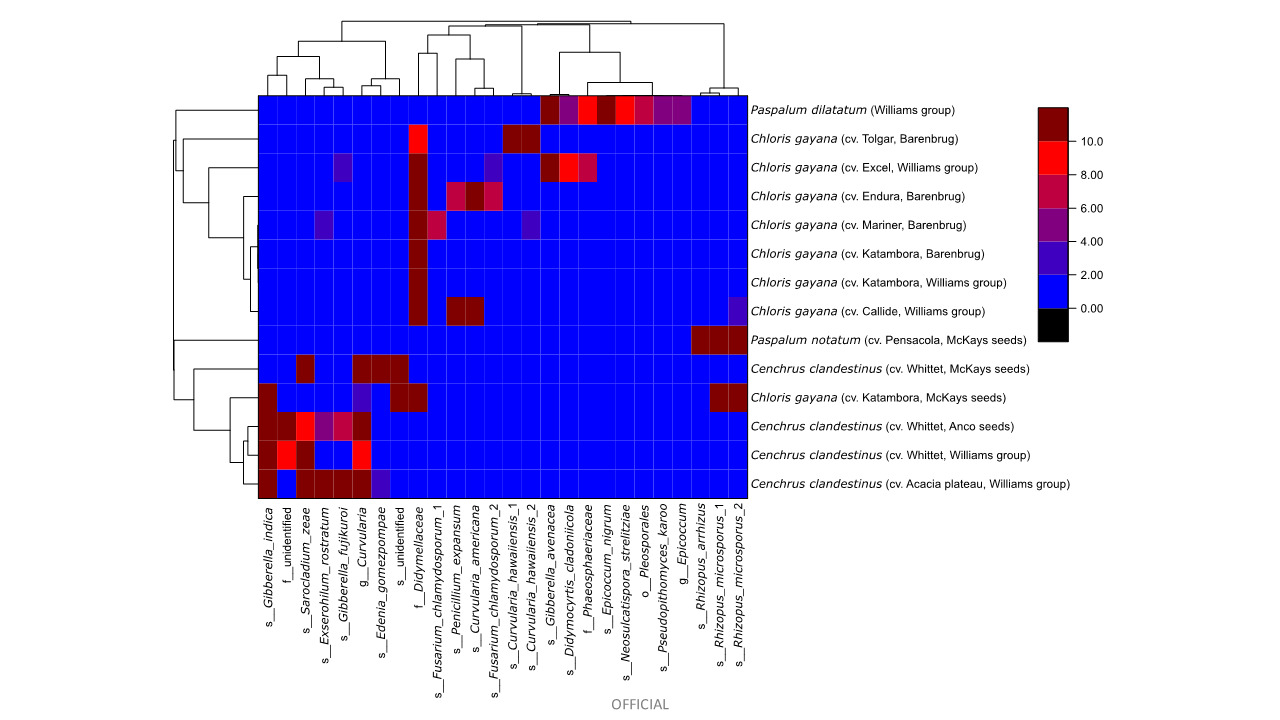

Supplement: Supplementary file 3 [file Image_3.tif]
